# 4439 Comparing the Impact of Adding an Educational Video Presentation to Universal Self-Consent for Remnant Clinical Biospecimens: A Single Blind Randomized-Control Trial

**DOI:** 10.1017/cts.2020.117

**Published:** 2020-07-29

**Authors:** Andrew Kyle, Stephanie E. Soares, Machelle D. Wilson, Nicholas R. Anderson, Javier E. Lopez

**Affiliations:** 1 University of California, Davis

## Abstract

OBJECTIVES/GOALS: BURRITO is an efficient strategy that provides full disclosure in the electronic medical record of a patient’s preference in real time. BURRITO uses printed materials only to inform patients and has a <50% rates of consent. We hypothesized that adding an informational video to the printed materials would increase donations. METHODS/STUDY POPULATION: This study was IRB-approved and was considered minimal risk. The BURRITO self-consent workflow process (Soares et. al, Biopreservation and Biobanking, IN PRINT) was developed in an outpatient cardiology clinic. In the same clinic, patients were randomized to receiving printed materials only (standard procedure) or the printed materials plus a 2.5-minute informational video (intervention) while waiting for the physician in the exam room. Randomization occurred at the level of the day in clinic. Patients were blinded to the nature of the study. Following the presentation of information, the patient’s decision on consent for donation was documented in the electronic record by ancillary clinical staff. Rates of consent were analyzed by a statistician not involved in the experiment and after completion of trial. RESULTS/ANTICIPATED RESULTS: Thirty-five clinic days were randomized to either intervention (17 days) or standard (18 days), and a total of 255 patients decided during their visit to either “opt-in” or “opt-out” to donating remnant biospecimens for future research. One hundred patients opted to defer deciding (28%). No significant demographic differences were noted between the study arms. The rate of consent was 73% vs. 58% in the intervention group and the control group, respectively (p-value = 0.014). This represents an increase in the odds of consenting with an informational video by 96% (OR = 1.96, 95% CI = 1.15 to 3.34). DISCUSSION/SIGNIFICANCE OF IMPACT: This is the first randomized trial to show that an informational video with printed materials is superior for when patients are self-consenting to opt-in for clinical remnant biospecimen donation. This result adds to the evidence that the BURRITO process plus video (BURRITOv) is an effective approach for biospecimen universal consenting.

